# Binding mode prediction of conformationally restricted anandamide analogs within the CB_1 _receptor

**DOI:** 10.1186/1750-2187-3-5

**Published:** 2008-02-26

**Authors:** Lea W Padgett, Allyn C Howlett, Joong-Youn Shim

**Affiliations:** 1 Neuroscience of Drug Abuse Research Program, Julius L. Chambers Biomedical/Biotechnology Research Institute, North Carolina Central University, Durham, NC 27707, USA; 2 Department of Chemistry and Physics, Armstrong Atlantic State University, Savannah, GA 31419, USA; 3 Department of Physiology and Pharmacology, Wake Forest University Health Sciences, Winston-Salem, NC 27157, USA

## Abstract

**Background:**

CB_1 _cannabinoid receptors are G-protein coupled receptors for endocannabinoids including anandamide and 2-arachidonoylglycerol. Because these arachidonic acid metabolites possess a 20-carbon polyene chain as the alkyl terminal moiety, they are highly flexible with the potential to adopt multiple biologically relevant conformations, particularly those in a bent form. To better understand the molecular interactions associated with binding and steric trigger mechanisms of receptor activation, a series of conformationally-restricted anandamide analogs having a wide range of affinity and efficacy were evaluated.

**Results:**

A CB_1 _receptor model was constructed to include the extracellular loops, particularly extracellular loop 2 which possesses an internal disulfide linkage. Using both *Glide *(Schrödinger) and *Affinity *(Accelrys) docking programs, binding conformations of six anandamide analogs were identified that conform to rules applicable to the potent, efficacious and stereoselective non-classical cannabinoid CP55244. Calculated binding energies of the optimum structures from both procedures correlated well with the reported binding affinity values. The most potent and efficacious of the ligands adopted conformations characterized by interactions with both the helix-3 lysine and hydrophobic residues that interact with CP55244. The other five compounds formed fewer or less energetically favorable interactions with these critical residues. The flexibility of the tested anandamide analogs, measured by torsion angles around the benzene as well as the stretch between side chain moieties, could contribute to the differences in ability to interact with the CB_1 _receptor.

**Conclusion:**

Analyses of multiple poses of conformationally-restricted anandamide analogs permitted identification of favored amino acid interactions within the CB_1 _receptor binding pocket. A ligand possessing both high affinity and cannabinoid agonist efficacy was able to interact with both polar and hydrophobic interaction sites utilized by the potent and efficacious non-classical cannabinoid CP55940. In contrast, other analogs characterized by reduced affinity or efficacy exhibited less favorable interactions with those key residues.

## Background

**Figure 1:**
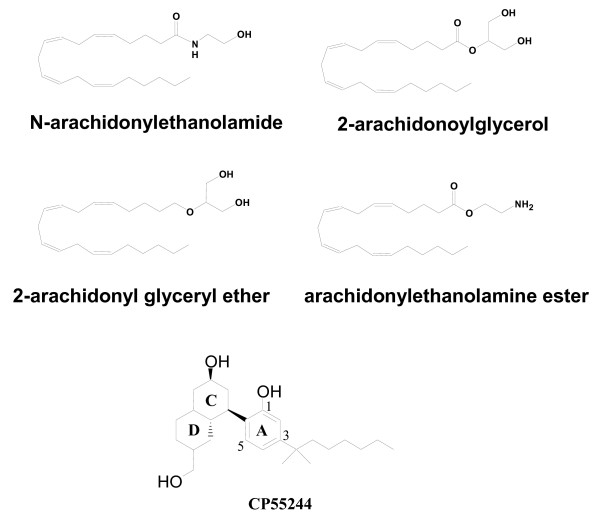
**Structures of Endocannabinoids and CP55244**. The structures of anandamide (*N*-arachidonylethanolamide), 2-arachidonoylglycerol (2-AG), nolandin ether (2-arachidonyl glyeryl ether, 2-AGE), and virodhamine (arachidonylethanolamine ester) are shown. The non-classical cannabinoid, CP55244 is depicted with the accepted numbering and ring nomenclature.

CB_1 _and CB_2 _cannabinoid receptors belong to the G-protein coupled receptor (GPCR) family. Members of this cell-surface receptor family are characterized by seven transmembrane (TM) helices connected by intra- and extracellular loops. Lipid ligands serve as the regulators of these receptors, and are collectively referred to as endocannabinoids. Anandamide (also known as *N*-arachidonylethanolamide) [1], 2-arachidonoylglycerol (2-AG) [2,3], 2-arachidonyl glyceryl ether (2-AGE or nolandin ether) [4], and virodhamine (arachidonyl ethanolamine ester) [5] (Fig. 1) have been reported to be endocannabinoids. A common feature of endocannabinoids is the long polyene hydrocarbon chain as the alkyl terminal moiety, which makes these compounds highly flexible with the potential to adopt many different binding conformations. Investigation of the bioactive conformation of anandamide can provide valuable insight into how endocannabinoids interact with the cannabinoid receptor and how their binding transfers the molecular signal to the coupled G-proteins. However, due to the conformational flexibility of anandamide, determination of the bioactive conformation within the CB_1 _receptor active site has been hampered. Although the binding mode of anandamide within the receptor remains to be determined, it has been demonstrated that anandamide is able to adopt quite different conformations in various media [6-8].

**Figure 2:**
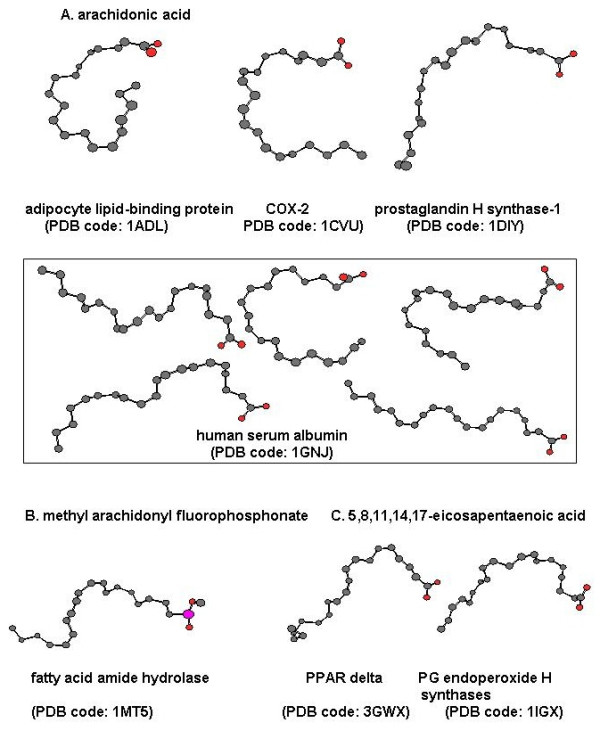
**Various conformations of arachidonyl derivatives in complex with proteins as found within the crystal structures of enzymes and binding proteins**. The National Library of Medicine Protein Structure Data Base was searched for structures of arachidonic acid and its metabolites and analogs determined from X-ray crystal structures of known enzymes and binding proteins. (**A**) Arachidonic acid in complex with adipocyte lipid binding protein (1ADL), cyclooxygenase active site of COX-2 (ICVU), prostaglandin H2 synthase-1 (1DIY), and human serum albumin (1GNJ); (**B**) Methyl arachidonyl fluorophosphonate in complex with fatty acid amide hydrolase (1MT5); (**C**) 5,8,11,14,17-Eicosapentaenoic acid in complex with peroxisome proliferator activated receptor delta (3GWX) and prostaglandin endoperoxide H synthase-1 (1IGX).

One theory for binding of ligands to the cannabinoid receptor suggests that anandamide is in an extended conformation, with the ethanolamide head group near the extracellular surface of the receptor and the long hydrocarbon chain extending down into the core of the membrane [9]. Another possibility is that anandamide may exhibit a binding mode similar to that demonstrated for arachidonic acid, its biological precursor. Arachidonic acid is known to be a substrate for oxygenase enzymes such as cyclooxygenases (COXs) and arachidonate 15-lipoxygenase (15-LO). X-ray crystal structures of arachidonic acid in a complex with these proteins revealed that arachidonic acid formed many different bioactive conformations, as shown in Fig. 2. Many X-ray structures for arachidonic acid show a bent conformation when co-crystallized in adipocyte lipid binding protein (PDB code: 1ADL), cyclooxygenase COX-2 (PDB code: 1CVU), prostaglandin H2 synthase-1 (PDB code: 1DIY), and fatty acid amide hydrolase (FAAH) (PDB code: 1MT5). Modeling studies of arachidonic acid in these proteins predict structures that are also bent in conformation [10-12]. Thus, it is fair to say that there may not be a single bioactive conformation of anandamide, but several multiple conformations may exist, particularly those in a bent form.

For the purpose of gaining insight into the binding of anandamide and its molecular interactions with the cannabinoid receptors, a series of conformationally-restricted anandamide analogs was synthesized and pharmacologically characterized [13]. In these restricted structures, aromatic rings replaced the long, unsaturated alkyl chains, based on a common pharmacophore model between endocannabinoids and traditional cannabinoids [14]. This class of anandamide analogs was designed based upon ligand-based modeling studies using constrained molecular dynamics simulations. A 3-dimentional quantitative structure-activity relationship (3D-QSAR) model was derived using constrained molecular field analysis (CoMFA) for a training set of 29 classical and nonclassical analogs, which rationalized the binding affinity in terms of steric and electrostatic properties. More importantly, this model predicted the potency of anandamide in excellent agreement with experimental data. In GTPγS binding assays to detect activation of G-proteins and adenylyl cyclase assays to detect effector regulation via Gi, compound **1 **(Table 1) was quite comparable in affinity as well as functional activity, whereas other compounds possessing small structural changes were significantly different in binding and/or activity [13].

**Table 1:** Structures and affinity and relative efficacy data for the conformationally restricted anandamide analogs

**Compound**	**Binding affinity, K**_i_**(nM)**	**GTPγS binding**	**Adenylyl cyclase**
CP55244	0.11	Strong stimulation	Strong inhibition
Anandamide	17	Strong stimulation	Strong inhibition
	38	Strong stimulation	Strong inhibition
	59	Moderate stimulation	Weak stimulation
	305	Moderate stimulation	Weak stimulation
	335	Strong stimulation	Moderate Inhibition
	371	Moderate stimulation	Weak stimulation
	4960	No change	Strong inhibition

Anandamide analogs were selected from a series of compounds developed and tested as reported previously [13]. Binding affinity was determined by the ability to compete for [^3^H]-CP55940 binding in rat brain membranes, G-protein activation was determined by the ability to stimulate [^35^S]-GTPγS binding to G-proteins in rat brain membranes, and effector activity was determined by the ability to regulate adenylyl cyclase activity (inhibit through Gi or stimulate through Gs) in purified membranes from N18TG2 cells.

In the present study, we report a docking study of compounds **1 **through **6 **(Table 1), using an updated model of the CB_1 _receptor in which the extracellular loops have been incorporated. Binding conformations of these ligands were modelled using two different docking packages: *Glide *(Schrödinger, Inc. Portland, OR) and *Affinity *(Accelrys, Inc. San Diego, CA). The working hypothesis was that these compounds should conform to the same binding rules as the very potent and stereoselective non-classical cannabinoid CP55244 [15] (Fig. 1). We describe the molecular interactions of these novel compounds with amino acid residues in the binding pocket of the CB_1 _receptor. The results provide insight into the properties of the ligand-receptor complex that are associated with affinity and efficacy.

## Results

### Extracellular loop 2 (E2) conformation of the CB_1 _receptor

**Figure 3:**
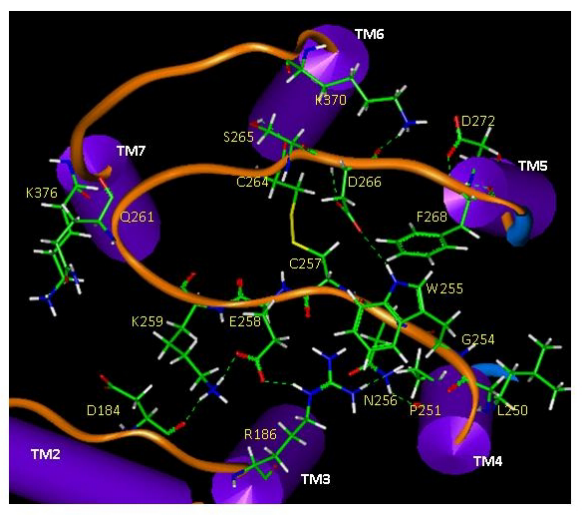
Extracellular loop placement on the human CB_1 _receptor model illustrating key interacting residues among E1, E2, and E3.

It was revealed from the present CB_1 _receptor model that extracellular loop 2 (E2) was located in the central region of the receptor on the extracellular side and packed at the entrance of the transmembrane core region (Fig. 3). Molecular dynamics (MD) simulations showed that conformations of E1 and E3 were influenced by the positioning of E2 (data not shown), suggesting that the extracellular loop region can readily adopt protean conformational changes in a membrane environment. As shown in Fig. 3, there were several non-bonding interactions between E1, E2, and E3 that might contribute to domain stabilization among the extracellular loops. For example, two H-bonds (R182/Q261 and D184/K259) between E2 and E1 and one H-bond (D266/K370) between E2 and E3 were identified. E2 itself was stabilized not only by the disulfide bridge (C257/C264), but also by H-bond (E258/K259) and aromatic stacking (W255/F268) interactions. A closer examination revealed that E2 was stabilized by two well-developed H-bond networks: 1) D184(E1)-K259(E2)-E258(E2)-R186(TM3)-N256(E2)-P251(TM4); and 2) W255(E2)-D266(E2)-K370(TM6) (Fig. 3). In addition, there were several H-bonds between E2 and TM residues, including G254(E2)-L250(TM4), F268(E2)-D272(TM6), and Q261(E2)-K376(TM7), which would contribute to the loop stabilization.

### Docking of the conformationally restricted anandamide analogs by *Glide*/*Prime*

**Figure 4:**
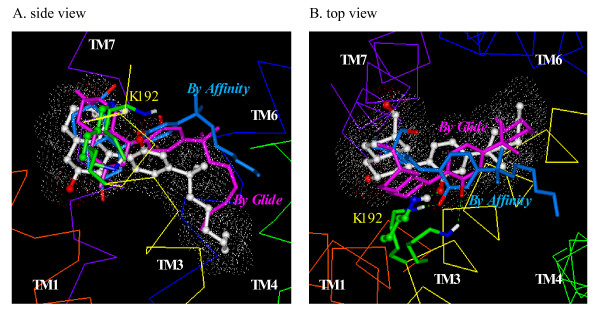
**Side (A) and top (B) views of the overlay of the docking modes of CP55244 determined using *Glide/Prime *(in lavender), *Affinity/SA *(in cyan) and the previously published CP55244 docking mode (in white)**. The CB_1 _receptor helical backbone is represented in Cα trace format. TM1 through TM7 are colored in red, orange, yellow, green, cyan, blue and purple, and the extracellular loops are colored in magenta.

As shown in Fig. 4, the CP55244 docking mode determined by *Glide/Prime *overlapped quite well with the previously published CP55244 docking model that had been developed using *Affinity*[16]. As described in [16], the putative binding conformation of CP55244 was identified and supported by a highly significant correlation between affinity determined from radioligand binding studies and ligand-receptor interaction energies for over 30 non-classical cannabinoid agonist ligands. In both models, the phenolic hydroxyl interacted with K3.28 (192). There was strong aromatic stacking of the A-ring with F7.35(379) which are 4.7 Å apart. This residue had contributed the greatest interaction in the previously published model [16]. The C3 tail interacts with numerous residues within TM3 and TM6, as was previously observed [16].

**Figure 5:**
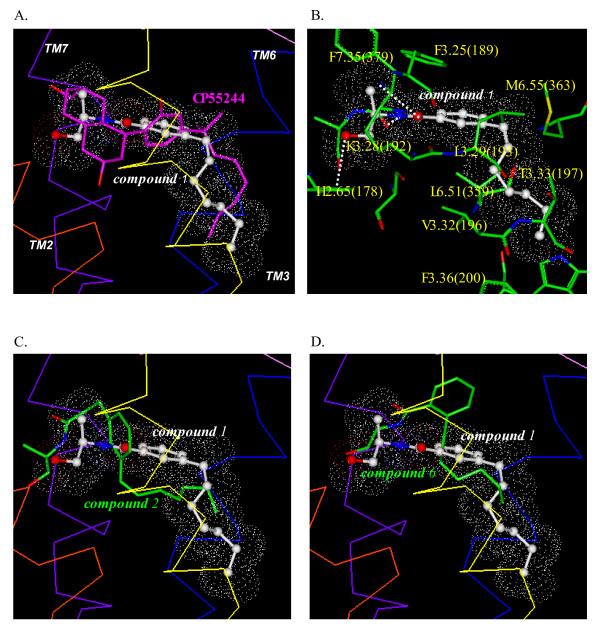
***Glide/Prime *models of CP55244 compound 1, 2 and 6**. **(A) **Overlay of the *Glide/Prime *models of CP55244 and compound **1**. **(B) **Key amino acid residues of the CB_1 _receptor for binding with the *Glide/Prime *model of compound **1**. H-bonding between compound **1 **and the binding pocket residues are represented with white dots. **(C) **Overlay of the *Glide/Prime *model of compound **2 **with compound **1**. **(D) **Overlay of the *Glide/Prime *model of compound **6 **with compound **1**.

In its optimum conformation within the binding pocket, compound **1 **occupies the same hydrophobic region of the receptor as that occupied by the C3 side chain of CP55244 (Fig. 5A). Compared to the model for CP55244, the aromatic ring of compound **1 **occupies the same location as the A-ring of CP55244, but is oriented nearly perpendicular to it. The amide side chain occupies the same location as the C/D fused cyclic region of CP55244, but is oriented in such a way that the compound **1 **terminal hydroxyl does not overlap with the D-ring hydroxyl of CP55244. There is potential for aromatic stacking between the compound **1 **aromatic ring and F3.25(189), which are 6.8 Å apart in this pose. Compound **1 **showed the presence of three hydrogen bonds: the amide NH with the backbone O of F7.35(379), the carbonyl oxygen of the amide with the side chain N of K3.28(192), and the terminal hydroxyl with the imidazole ring N of H2.65(178) (Fig. 5B). Compounds **1 **and **2**, which differ in the linker that separates the amide moiety from the aromatic ring, have the greatest affinity (Ki = 38 nM and 59 nM, respectively) for the receptor (see Table 1). In the best *Glide/Prime *pose for compound **2**, the benzene ring was perpendicular to the position of the aromatic ring in compound **1 **as a result of the considerable aromatic stacking with F2.61(174), F2.64(177) and F3.25(189) (Fig. 5C). Compared with compound **1**, compound **2 **partially occupied the hydrophobic pocket and formed H-bonds between the amide O and the side chain N of K7.32(376) and between the amide N and the side chain hydroxyl O of S7.39(383), but no H-bond with K3.28(192). Compound **3 **which lacks the methyl group of compound **2**, exhibited limited hydrophobic interaction with neighboring residues such as I1.35(119) and M7.40(383) (data not shown). Compound **4**, which lacks the methyl group of compound **1**, was raised up toward the extracellular surface compared with the position of the aromatic ring in compound **1**, such that the alkyl tail of **4 **could not extend as deeply into the pocket (data not shown). Comparisons of the docking of compounds **5 **and **6 **with **1 **indicate that the *ortho*-substituted aromatic ring was severely displaced toward the extracellular surface and TM2 and TM3, and the heptenyl tail failed to occupy the hydrophobic pocket (Fig. 5D). The degrees of interaction with the hydrophobic pocket for compounds **1**, **2, 5 **and **6 **were estimated by a root mean square deviation (RMSD) from the corresponding carbons of CP55244. The RMSD for the six tail carbons were 2.8 Å, 4.8 Å, 4.6 Å and 5.4 Å, respectively.

### Docking of the conformationally restricted anandamide analogs by *Affinity*/*SA*

The *Affinity/SA *model of CP55244 overlaps with the previously published docking model [15,16], as shown in Fig. 4 in which the CP55244 phenolic hydroxyl formed an H-bond with K3.28(192). It is interesting to note that the C3 side chain in this model pointed toward the hydrophobic pocket region between TM3 through TM5 (Fig. 4B). This region is somewhat different from the proposed hydrophobic pocket for the previously published CP55244 model, in which the C3 side chain tail pointed toward the TM core region between TM3 and TM6. The optimum *Affinity/SA *model of CP55244 shows strong aromatic interaction with F7.35(379), as shown for the *Glide/Prime *model of CP55244 and for the previously published CP55244 model [16]. Aromatic stacking interactions exist between the A-ring and F3.25(189), which are 6.1 Å apart, and between the A-ring and F7.35(379), which are 5.4 Å apart

**Figure 6:**
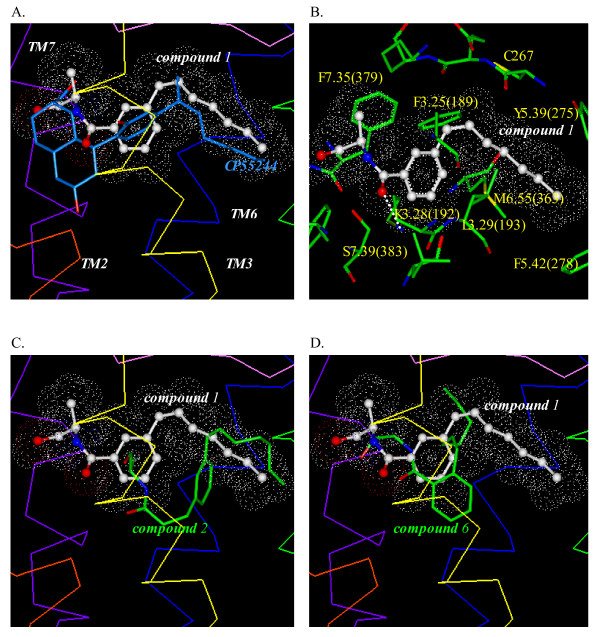
***Affinity/SA *models of CP55244 and compound 1, 2 and 6**. **(A) **Overlay of the *Affinity/SA *models of CP55244 and compound **1**. **(B) **Key amino acid residues of the CB_1 _receptor for binding with the *Affinity/SA *model of compound **1**. H-bonding between compound **1 **and the binding pocket residues is represented with white dots. (**C**) Overlay of the *Affinity/SA *model of compound **2 **with compound **1**. (**D**) Overlay of the *Affinity/SA *model of compound **6 **with compound **1**.

As shown in Fig. 6A, the optimum fit of compound **1 **is comparable to that occupied by CP55244, including the co-localization of the hydrophobic side chains of both molecules, and the aromatic ring of compound **1 **with the A-ring of CP55244. Compound **1 **(Fig. 6B) exhibits a H-bond between K3.28(192) and the carbonyl of the amide. The compound **1 **terminal hydroxyl occupies the same location as the D-ring hydroxyl of CP55244. The superposition model of CP55244 and compound **1 **suggests that the weaker binding affinity of compound **1 **compared with CP55244 could be due to the missing moiety in compound **1 **that could correspond to the C/D-ring moiety of CP55244. There is potential for aromatic stacking between the compound **1 **aromatic ring and F3.25(189) as well as F7.35(379) as can be seen in the Compound **1 **movie (Additional File 1). Compound **2 **exhibited a greater variety of binding modes compared with compound **1**. The pose shown in Fig. 6C lacked the H-bonding interaction with K3.28(192), although the C3 side chain occupied the same hydrophobic pocket as compound **1 **and exhibited energetically favorable aromatic stacking with F5.42(278), which are 6.7 Å apart. Poses in which compound **2 **exhibited H-bonding with K3.28(192) showed that the aromatic ring of compound **2 **was displaced from the position of the aromatic ring in compound **1 **(see Compound **2 **movie (Additional File 2)). Compound **5 **occupied an almost identical region as compound **1**; in particular, its alkyl tail occupied the same region. It also exhibited a set of H-bonding interactions between the aromatic ring OH and K3.28(192), and between the terminal OH and S7.39(383). The aromatic ring of compound **5**, which is 5.5 Å apart from F7.35(379), was skewed compared with compound **1 **due to the presence of two carbon linker atoms between the aromatic ring and the amide moiety (not shown). Compound **6 **showed H-bonding interactions for the amide and OH terminal with K3.28(192) and S7.39(383), as did compound **5 **(data not shown). However, as a result of the reduced flexibility imposed by the *ortho *arrangement of the moieties on the ring (see below), the heptenyl tail moiety was severely restricted, thereby limiting interaction with the hydrophobic residues (Fig. 6D) (see Compound **6 **movie (Additional File 3)). The RMSD, an estimate of the degree of interaction with the hydrophobic pocket, for the six tail carbons of compounds 1, 2, 5 and 6, when compared with CP55244, as were 2.2 Å, 2.7 Å, 0.9 Å and 7.2 Å, respectively.

### Linear Interaction Energy (LIE) binding free energy

**Figure 7:**
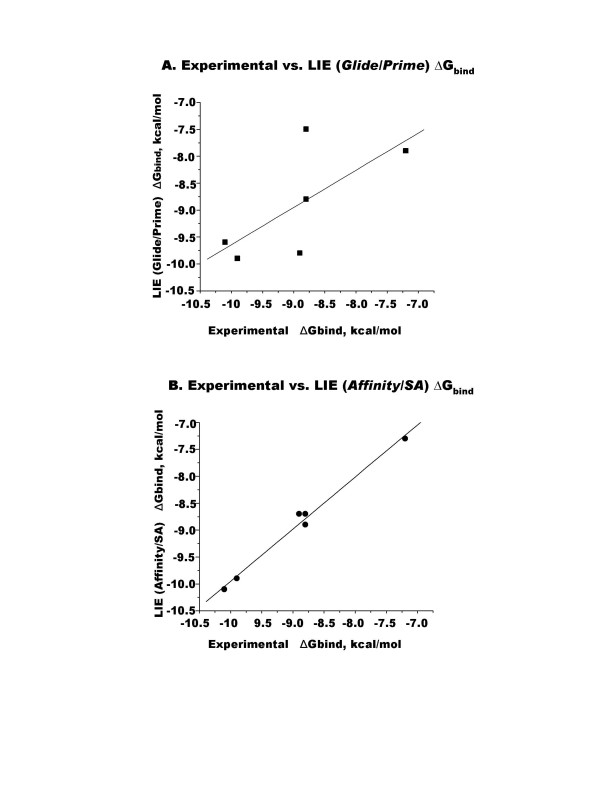
Binding energy correlation between experimental ΔG_bind _and LIE (*Glide/Prime*) ΔG_bind _(A) or LIE (*Affinity/SA*) ΔG_bind _(B).

In order to validate the optimum docking conformations by a means that would consider the solvation and entropy effects, we employed the Linear Interaction Energy (LIE) method based on the Surface Generalized Born continuum solvation model [17]. The experimental Δ*G*_bind _values determined from the binding affinity data were compared with the estimated Δ*G*_bind _values obtained from the identified docking conformations. Fitting the estimated Δ*G*_bind _values from either the *Glide/Prime *conformations or the *Affinity/SA *conformations to their experimental Δ*G*_bind _values yielded the following LIE equations: for the *Glide/Prime *conformations, Δ*G*_bind _= 0.381<ΔU_vdw_> + 0.028 <ΔU_elec_> + 1.271 ΔSASA; and for the *Affinity/SA *conformations, Δ*G*_bind _= 0.348 <ΔU_vdw_> + 0.032 <ΔU_elec_> + 1.084 ΔSASA. For both conformations, the RMSD of <1.0 kcal/mol was obtained. Using these equations, the calculated Δ*G*_bind _values correlated well with the experimental Δ*G*_bind _values for compounds **1 **through **6 **(Fig. 7 and Table 2). The Jackknife correlation coefficient indicated that the *Affinity/SA *conformation derived LIE equation is more robust than the *Glide/Prime *conformation-derived LIE equation (Table 2). However, these data do not predict efficacy of ligand-induced conformational changes that may be required for biological activity.

**Table 2:** Binding free energy comparison between the experimental and LIE (*Glide/Prime*) or LIE (*Affinity/SA*) values for compounds 1 through 6

	**Δ*G*_bind_(kcal/mol)**		
	
**Compound**	**LIE (*Glide/Prime*)**	**LIE (*Affinity/SA*)**	**Experimental**
**1**	**-9.6**	**-10.1**	**-10.1**
**2**	**-9.9**	**-9.9**	**-9.9**
**3**	**-9.8**	**-8.7**	**-8.9**
**4**	**-8.8**	**-8.7**	**-8.8**
**5**	**-7.5**	**-8.9**	**-8.8**
**6**	**-7.9**	**-7.3**	**-7.2**

The Glide/Prime LIE fit was determined using the following equation with a RMSD of 0.6 kcal/mol: Δ*G*_bind _= 0.381<ΔU_vdw_> + 0.028<ΔU_elec_> + 1.271ΔSASA. The Affinity/SA LIE fit was determined using the following equation with a RMSD of 0.1 kcal/mol: Δ*G*_bind _= 0.348<ΔU_vdw_> + 0.032<ΔU_elec_> + 1.084ΔSASA. The Experimental Δ*G*_bind _values were calculated from the experimental *K*_i _values (see Table 1) assumed to be equivalent to *K*_D_, using Δ*G*_bind _= RT ln *K*_i _for T = 298 K.

Table 3 represents the ligand-receptor interactions within the hydrophobic pocket that were identified for CP55244 using *Affinity/SA *(see Fig. 4). Those binding pocket residues within 3.5 Å of the C3 tail of the ligand were: F3.25(189), L3.29(193), T3.33(197), P4.60(251), F5.42(278), and M6.55(363). In addition to these residues, several residues from E2 and F7.35(379) near the entrance of the hydrophobic pocket appeared to be close enough to interact with the C3 tail of the ligand. As shown in Table 3, of the five different poses tested for compound **1**, most showed binding interactions with more than five of these hydrophobic pocket residues. Compound **2 **exhibited three to four such interactions. Compound **6**, the least potent compound tested, showed many fewer interactions with these residues and smaller interaction energies. The binding interaction with L3.29(193) was conserved for all of the ligands, indicating that this binding interaction might be of particular importance to the overall binding mode. On the other hand, the binding interactions with Y5.39(275), F5.42(278) and M6.55(363) were maintained for all poses of compound **1**, but only some poses of compound **2**, indicating that these interactions might be important for the trigger mechanism of receptor activation [15]. It is interesting to note that there were additional H-bonding interactions between the terminal hydroxyl of the ligand and E(258) or S7.39(383), particularly for compounds **2 **and **6**. These H-bonding interactions may distort the conformations of these compounds thereby reducing the efficacy associated with activation. These data indicate that the hydrophobic pocket is crucial for ligand binding, and support the proposal that the degree of interaction with these hydrophobic pocket residues is a key determinant in ligand binding affinity and efficacy.

**Table 3:** Ligand-receptor non-bonding and H-bonding interactions identified for CP55244 by *Affinity *and compared with compounds 1, 2, and 6.

**compound**	nonbonding interaction energy (kcal/mol)										
		Key Binding Site Residues within 3.5 Å of CP55244 hydrophobic pocket							Potential H-bond forming residues		

		F3.25 (189)	L3.29 (193)	T3.33 (197)	P4.60 (251)	Y5.39 (275)	F5.42 (278)	M6.55 (363)	K3.28 (192)	S7.39 (383)	E (258)

**CP55244**	Coulombic van der Waals Total	-0.06 -1.20 -1.26	0.06 -2.21 -2.15	0.02 -0.64 -0.62	0.00 -0.37 -0.37	0.00 -1.20 -1.20	0.00 -0.79 -0.79	0.00 -0.44 -0.44	-0.88 -0.54 -1.41		

**compound 1**											

Pose1	Coulombic van der Waals Total	0.00 -1.33 -1.33	0.00 -1.64 -1.64			0.00 -0.74 -0.74	0.00 -0.49 -0.49	-0.02 -0.75 -0.77	-1.19 -0.13 -1.32		
Pose2	Coulombic van der Waals Total	0.01 -1.58 -1.57	0.00 -1.79 -1.79	-0.01 -0.35 -0.36		0.00 -0.46 -0.46	-0.01 -0.45 -0.46	-0.02 -0.74 -0.76	-1.14 -0.59 -1.73		
Pose3	Coulombic van der Waals Total		-0.01 -2.11 -2.12	0.20 -0.75 -0.55	0.01 -0.45 -0.44	0.09 -1.09 -1.00	0.00 -0.51 -0.51	0.01 -0.78 -0.77	-1.33 -0.74 -2.07	-0.53 -0.83 -1.36	
Pose4	Coulombic van der Waals Total		-0.02 -1.55 -1.57	0.00 -0.19 -0.19		0.01 -0.57 -0.56	0.01 -0.48 -0.47	0.02 -0.87 -0.85	-1.21 -0.60 -1.81		
Pose5	Coulombic van der Waals Total	-0.01 -1.32 -1.33	-0.02 -1.85 -1.87	0.00 -0.83 -0.83	0.00 -0.29 -0.29	0.38 -0.90 -0.52	0.00 -0.43 -0.43	-0.02 -1.01 -1.03	-2.39 0.09 -2.30	-0.20 -1.15 -1.35	

**compound 2**											

Pose1	Coulombic van der Waals Total		-0.02 -1.48 -1.50	0.00 0.02 0.02		0.00 -0.37 -0.37		-0.01 -0.84 -0.85	-1.39 -0.88 -2.26		-0.71 0.06 -0.65
Pose2	Coulombic van der Waals Total			0.01 -0.91 -0.90		-0.01 -0.06 -0.07		-0.03 -1.01 -1.04	-0.98 -0.66 -1.64		-0.63 -0.61 -1.24
Pose3	Coulombic van der Waals Total		-0.04 -1.67 -1.71	-0.03 -0.75 -0.78			0.00 -0.67 -0.67	-0.02 -1.22 -1.24	-1.33 -0.48 -1.81		
Pose4	Coulombic van der Waals Total		0.07 -2.85 -2.79		0.00 -0.29 -0.29		0.01 -0.91 -0.90				
Pose5	Coulombic van der Waals Total	0.05 -0.77 -0.72	0.02 -1.58 -1.56					-0.01 -1.21 -1.22	-1.16 -0.51 -1.67		-0.65 -0.25 -0.90

**compound 6**											

Pose1	Coulombic van der Waals Total	-0.01 -0.88 -0.89	-0.02 -1.52 -1.54			0.00 -0.09 -0.09		-0.01 -0.91 -0.92	-2.29 -0.51 -2.80		
Pose2	Coulombic van der Waals Total	0.01 -1.12 -1.11	-0.04 -1.76 -1.80	-0.01 -0.45 -0.46				0.00 -0.89 -0.89	-2.22 -1.06 -3.28		
Pose3	Coulombic van der Waals Total	0.04 -0.89 -0.85	0.00 -1.31 -1.31					0.00 -1.04 -1.04	-1.23 -0.55 -1.78	-0.39 -1.41 -1.80	
Pose4	Coulombic van der Waals Total	0.02 -0.76 -0.74	0.00 -1.39 -1.39					-0.01 -0.77 -0.78	-1.16 -0.47 -1.63	-0.52 -1.23 -1.75	
Pose5	Coulombic van der Waals Total	0.06 -0.93 -0.87	0.00 -1.36 -1.36					0.00 -0.94 -0.94	-1.06 -0.57 -1.64	-0.46 -0.92 -1.38	

### Aromatic stacking by multiple copy simultaneous search (MCSS)

**Figure 8:**
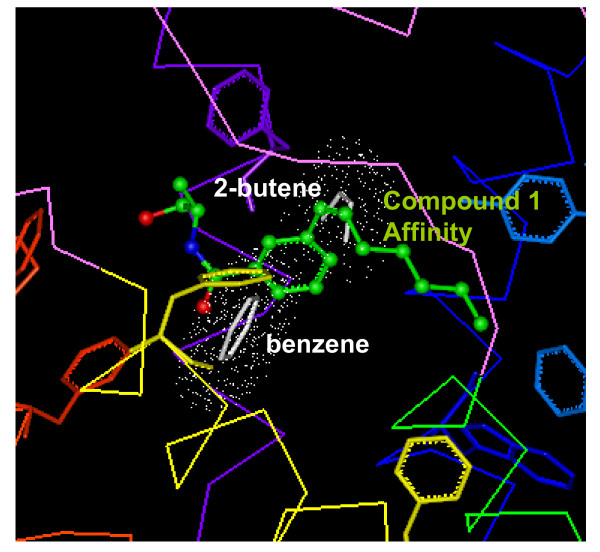
**Comparison of the best position of the benzene and *cis*-2-butene moieties from *MCSS *with the *Affinity/SA *model of compound 1**. Aromatic residues at or around the ligand binding pocket are represented including F3.25(189) and F7.35(379) identified as potentially important residues for aromatic stacking with compound **1**.

Aromatic stacking appears to be important for the ligand binding to CB_1_, as exemplified in all the potent ligand compounds that contain at least one aromatic ring (i.e., the A-ring of cannabinoids, and the indole ring of WIN55212 and other aminoalkylindole analogs) [18,19]. To further validate the docking position of the benzene ring in the tested compounds, we explored the best position of two key molecular fragments of compound **1 **using the multiple copy simultaneous search (*MCSS*), a tool implemented in *InsightII *that predicts potential ligand binding sites. As shown in Fig. 8, the position of the benzene ring nearly overlaps with the aromatic ring of compound **1**. The benzene ring in its lowest energy position was located such that many aromatic residues (including: F3.25(189), 5.80 Å; F2.61(174), 6.16 Å; F7.35(379), 8.06 Å) were potentially available to contribute to the aromatic-aromatic interaction within the binding pocket. The lowest energy position of the *cis*-2-butenyl moiety shared π-π interactions with three aromatic residues: F3.25(189), Y5.39(275), and F7.35(379), and the *cis*-2-butenyl moiety was quite close to the position of the vinyl moiety of compound **1 **(Fig. 8). It can be postulated that the lowest energy position of the *cis*-2-butenyl moiety would be equivalent to the hydrophobic pocket that was proposed to be crucial in cannabinoid binding [18], at least for those compounds containing the unsaturated hydrocarbon side chain for which the π-π interaction would be the principal interaction [19].

### Conformational flexibility explored by MD simulations

**Figure 9:**
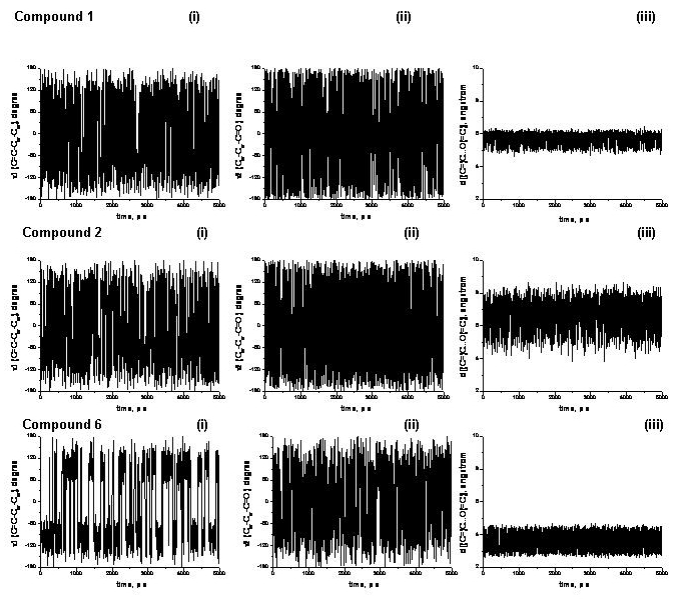
**Ligand flexibility estimated from MD simulations for compounds 1, 2 and 6**. The torsion angles around the aromatic ring, defined as τ 1(C = C-C_ar_-C_ar_) and τ 2(C_ar_-C_ar_-C = O), and the distance between the first carbon atom of the 1-heptenyl tail and the amide oxygen atom, defined as d [(C =)C...O(= C)] during a 5 ns MD simulation of compounds **1**, **2**, and **6**. To resemble the highly hydrophobic environment within the binding pocket, the dielectric constant ε of 4.0 was used for the electrostatic interaction energy.

In studies to explore the flexibility of the conformationally-restricted anandamide analogs, we examined torsion angles around the aromatic ring, defined as τ1(C = C-C_ar_-C_ar_) and τ 2(C_ar_-C_ar_-C = O) during a 5 ns MD simulation of compounds **1**, **2**, and **6 **(Fig. 9). It was assumed that the τ 1 torsion angle would indicate the flexibility of the 1-heptenyl tail of the ligand, while the τ 2 torsion angle would indicate the flexibility of the polar amide moiety of the ligand. As shown by the torsion angle of τ 1, compounds **1 **and **2 **appeared to be quite flexible around the aromatic ring. In contrast, compound **6 **appeared to be much less flexible as indicated by having only two predominant orientations, around 120 and -120 degrees, respectively. The less flexible τ 1 torsion angle of compound **6 **would lead to poor binding interaction with the hydrophobic binding pocket residues. Interestingly, compound **6 **showed quite diverse τ 2 torsion angle values, comparable to those of compounds **1 **and **2**, suggesting that the polar amide moiety exhibited great flexibility in the molecules of this series, and could occupy alternative positions within the binding pocket.

In Fig. 9, the distance d [(C =)C...O(= C)] between the first carbon of the 1-heptenyl tail and the amide oxygen was monitored as the MD simulation progressed. The distance d [(C =)C...O(= C)] showed quite interesting differences among compounds **1**, **2**, and **6**. For compound **1**, this distance remained in a very narrow range of values between 5 Å to 6 Å throughout the simulation. As a reference, the corresponding distance for the best docking conformation of compound **1 **was 5.9 Å, and the distance between the first carbon of the C3 tail and the A-ring hydroxyl oxygen of CP55244, as found in the best docking conformation, was 5.0 Å. Compared with compound **1**, compound **2 **showed a rather diverse range (4 Å to 8 Å) of the d distance, suggesting that compound **2 **might not be efficient at occupying both the hydrophobic pocket and maintaining an interaction with K3.28(192) at the same time for the best conformational changes associated with efficacy. In contrast, compound **6 **showed a narrow range of the distance d values (3 Å to 4 Å). This restricted distance might be too limited to satisfy the two important binding interactions with residues in the hydrophobic pocket as well as the interaction with K3.28(192).

## Discussion

Several CB_1 _receptor studies support the importance of E2 for cannabinoid ligand binding and efficacy [20-23]. For many rhodopsin family GPCRs, a disulfide linkage between Cys residues from TM3 and E2 is important for maintaining receptor structure and function [24,24-27], possibly stabilizing coupling between the extracellular loop and TM core domains [28]. Because the CB_1 _receptor lacks such a disulfide linkage, evidence implicates the formation of an alternative disulfide linkage between the two E2 Cys residues (i.e., C257 and C264) in E2 [20,21]. Our model suggests that E2 could be further stabilized by a set of H-bonding networks and other non-bonding interactions with E1, E3 and TM domain residues. A recent mutation study [29] suggests that E1 is indirectly involved in modulating ligand binding through interaction with the neighboring E2, and that the negatively charged D3.20(184) would interact with the K259 residue in E2. In agreement, our receptor model shows that E1 and E2 interact closely through H-bonding and/or salt bridge formation (R182/Q261 and D184/K259) (Fig. 3). The possibility that K183 in E1 interacts with E258 would be less convincing due to the following reasons: (1) the K183A mutation was less detrimental than the D3.20(184)A mutation in CP55940 binding [29]; and (2) rat and mouse CB_1_, in which the E258 residue in E2 is replaced by a Lys, is incapable of such ionic interaction but still is comparable to the human CB_1 _receptor for CP55940 binding [30,31]. One of the H-bonding networks (i.e., W255(E2)-D266(E2)-K370 (TM6)), and an aromatic stacking interaction of W255 and F268, appear to function as a lock such that E2 is kept in a closed conformation (see Fig. 3). It can be postulated that as the receptor environment changes due to the approaching ligand, this lock might be disrupted, leading to an open conformation of E2 with enhanced mobility, together with a transiently reduced E2 interaction with the neighboring segments. This would create a space for the incoming ligand to enter the binding site from the extracellular side.

The effect of solvent was not rigorously considered under the assumption that E2 would exist as close as possible to the entrance of the TM core pocket as suggested from the X-ray structure of rhodopsin [32]. Our models favor the E2 conformers with increased interactions with the receptor residues for packing. Calculations of MD simulations in explicit water suggested that inclusion of solvent would not have much effect on the E2 conformation, inasmuch as the RMSD was 1.19 Å when compared with the present receptor model. However, the receptor/membrane environment, wherein the membrane polar head groups as well as the extracellular receptor residues greatly exert their influence, would be important for precisely determining the E2 conformation [33].

Assuming that the 1-heptenyl moiety of the structurally-restricted anandamide analogs would be equivalent to the 1',1'-dimethylheptyl of cannabinol analogs [19,34] and that the ethanolamide moiety is needed to form H-bonding to K3.28(192), we determined the docking poses of compounds **1 **through **6**. At first glance, compounds **1 **through **6 **look similar in structure in that they commonly contain a benzene ring, the 1-heptenyl group, and hydroxylamide (see Table 1). However, they significantly differ conformationally due to the difference in position of the substituent on the aromatic ring (i.e., *ortho*-versus *meta*-) and the difference in the number of carbon atoms linking the hydroxylamide moiety to the benzene ring (i.e., the linker size). Both *Glide *and *Affinity/SA *approaches, as any other docking program, attempt to sample as many ligand conformations as possible within the predefined binding pocket. However, due to the enormous conformational flexibility of the tested compounds, the currently applied docking methods appear to be limited in properly sampling the conformation space. In *Glide*, the definition of the computational search space, based upon defining a box around the ligand in its initial placement into the receptor complex, is a fairly conservative approach that did not generate as wide a sampling of poses as the *Affinity/SA *process. *Glide *does not move the protein side chains, possibly preventing the escape of the ligand from a local energy minimum. As a result, the poses from *Glide *are more predisposed to have a docking conformation similar to that of CP55244, which was used as a model for the initial point of the simulation. In an effort to increase the search space as well as to optimize the conformations, the poses were run through a sequence of *Glide *and *Prime *steps. The *Affinity/SA *method permits the opportunity for the system to overcome local energy traps, and appears to be better in conformation sampling. Each approach uses different scoring functions: the *Glide *score [35] in *Glide *versus binding interaction energy in *Affinity/SA*. One difficulty in scoring docking conformations of the tested compounds derives from the generation of many local energy minima conformations without substantial energy differences. Thus, we applied these scoring functions to screen a set of conformations for each compound for which the LIE method was applied to select the best docking conformations. The different positions of the K3.28(192) residue and the different hydrophobic pockets determined by *Glide *and *Affinity/SA *exemplifies and emphasizes the notion of multiple micro-conformational states for the receptor-ligand complex [36]. With the TM backbone fixed, the side chains of the binding pocket residues as well as the extracellular loop residues were allowed to move freely during the docking simulations. Because the binding pocket within the TM core is relatively large, it is possible for the pocket residues to contribute to the modification of the pocket shape. This is especially true for hydrophobic residues, which can only be held relatively fixed when they have close contact for favorable interactions with neighboring hydrophobic residues. In contrast, polar residues that form very specific H-bonds and/or salt bridges to other polar residues or the amide backbone appear to be important for helical packing to stabilize the receptor framework structure.

In our previous analyses of the steric trigger mechanism of CB_1 _receptor conformational changes induced by agonists [15], the efficacy of non-classical cannabinoid CP55244 was explored by conformational analysis, rotational barrier calculations, and molecular dynamics (MD) simulations. It was demonstrated that the torsion angles of the C3 side chain showed the most dramatic change when compared with the ground-state receptor-bound conformation, indicating that rotation around these torsion angles could be responsible for releasing the ligand strain energy. We also demonstrated that multiple stages were involved in the ligand conformational change. We proposed that the C3 side chain serves as the steric trigger, while the ACD-ring moiety of CP55244 serves as the plug, and that steric clash with helices within the binding pocket could induce microconformational adaptation within the protein. This mechanism would suggest that rotational flexibility in a ligand may be as important a determinant of agonist activity as the pharmacophoric elements that can be identified. Compound **1 **exhibits the best activity of the compounds tested, showing greater than two-fold increase over basal in [^35^S]GTPγS binding to G-proteins and maximal efficacy in the Gi-mediated inhibition of adenylyl cyclase. The anchoring of the amide moiety by multiple H-bonds as well as the stable positioning of the aromatic ring by interactions with hydrophobic residues constitutes the plug, consistent with a steric trigger mechanism of activation focusing on conformational mobility of the alkyl chain. This is similar to the C/D-ring region of CP55244 being anchored, and the dimethylheptyl chain being capable of free rotation around its single bonds, possibly inducing conformational changes in the receptor upon rotation [15]. The importance of the ligand interaction with the receptor through the hydrophobic tail is exemplified in the receptor binding properties of CP55940 [37] which exhibited a Ki = 0.137 nM. The analog having no A-ring OH to H-bond with K3.28(192) exhibited a Ki = 40.2 nM, whereas the analog having no C3 tail, exhibited a Ki = 441 nM, representing a ten-fold greater loss of affinity.

Among tested anandamide analogs, compound **2 **shows quite similar binding affinity as anandamide (see Table 1). However, it is striking that compound **2 **fails to stimulate GTPγS binding or to inhibit adenylyl cyclase activity (see Table 1). This would be expected of a compound that either could not form a "plug" or could not contribute a steric trigger interaction to promote a conformational change associated with receptor activation. As suggested from our MD simulation (see Fig. 9B), compound **2 **might not be efficient at occupying both the hydrophobic pocket and maintaining an interaction with K3.28(192) simultaneously. This would reduce the probability of conformational changes associated with efficacy, rendering the molecule less likely to trigger a conformational change in the hydrophobic pocket. In agreement with this observation, the least potent of the series, compound **6**, shows the least fit to the hydrophobic pocket (Figs. 5 &6). As shown in the compound **6 **movie (Additional File 3), compound **6 **does not conform well to the space occupied by CP55244 and compound **1**. It appears that the very poor binding affinity of compound **6 **is due to the lack of interaction with the key binding site amino acid residues that are involved in binding to CP55940 or compound **1**. In agreement, our MD simulation suggested that compound **6 **(see Fig. 9C) would fail to exhibit geometry required to satisfy the two important binding interactions with residues in the hydrophobic pocket as well as the interaction with K3.28(192). In addition, an increased conformational rigidity of the tail of this compound suggests its reduced efficacy as the steric trigger.

## Conclusion

Anandamide can adopt many diverse conformations, analogous to arachidonic acid which, as a substrate for proteins, interacts via different bioactive conformations. Thus, as we identified by two computational methods for the conformationally-restricted anandamide analogs in the present study, the docking of anandamide within the CB_1 _active site could be presumed to interact in such a way that includes H-bonding to K3.28(192) and occupancy within the hydrophobic pocket. The steric trigger mechanism of ligand induction of receptor activation demands that both a stable "plug" and a movable "trigger" be secured by receptor-ligand interactions in order to achieve high efficacy. Due to the absence of either plug or trigger, compounds having poor efficacy would not be able to induce a micro-conformational modification in the binding pocket that could initiate receptor activation.

## Methods

### Computational Methods

All computations and molecular modeling were carried out on a Silicon Graphics Origin 2000 or Origin 350 workstation.

### Construction of the CB_1 _receptor with the extracellular loops incorporated

As the starting structure, the transmembrane helical domain (TM) of the human CB_1 _receptor was constructed using the X-ray structure of rhodopsin (PDB code: 1L9H) [32] as a template, and the E1 and E3 were constructed by the random loop search method implemented in *InsightII *(version 2000, Accelrys Inc. San Diego, CA). The E2 (G254-W-N-C-E-K-L-Q-S-V-C-S-D-I-F-P269) was designed with the formation of a disulfide linkage between the two Cys residues (i.e., C257 and C264) in E2, as supported by recent studies [20,21]. Assuming the formation of an eight-membered ring through such a disulfide linkage, the homologous amino acid sequences to these residues whose structures were determined experimentally were identified from DSDBASE [38]. Among 20 cyclic peptides that showed >70% sequence homology, those structures were discarded that were from Cys-rich proteins or exhibited poor geometry to fit into the TM core. The remaining candidate cyclic peptides were docked into the extracellular pore region of the CB_1 _receptor model by using ZDOCK [39,40]. To select a reasonable position of the peptide at the extracellular side of the TM core region, the following criteria were considered: 1) the distance between the Cα of C257 and the Cα of the C-terminal residue of TM4, L4.62(253) was set to be <10 Å with the presence of the short linking residues (GWN); and 2) interactions with F3.25(189) were favored because of evidence that the interaction of this residue with E2 is critical for CP55940 binding [29]. After determining plausible orientations of the cyclic peptide fragment, the remaining distal and marginal residues of E2 were introduced by the loop search approach implemented in *InsightII*. The simulated annealing (SA) procedure was then performed with the TM backbones constrained, the ω angles held to 180 degrees, and the φ and ψ angles of the residues forming the cyclic peptide fragment held constant at the values from the X-ray structure. The protocol for SA involved heating to 2,000 K in steps of 100 K over 20 ps, a holding time at 2,000 K for 10 ps, followed by cooling to 300 K over 34 ps. After holding at 300 K for 10 ps, minimization was performed for 2,500 iterations or until 0.01 kcal/molÅ energy gradient convergence. The final minimized conformer was saved and at the same time used for the next cycle of the SA run. By repeating this procedure, approximately 1000 conformers were obtained, which were analyzed by a combination of *Profiles-3D *scores and molecular mechanics energies criteria [41]. The best CB_1 _receptor model was selected and used for the following docking simulations of the rigid anandamide analogs. For energy minimizations and SA simulations, the cell multipole method with a dielectric constant (ε = 4.0) was used for summation of non-bonding interactions.

### Docking of the ligands

Compounds **1 **through **6 **were docked to the CB_1 _receptor using *Affinity *and *Glide*. CP55244 was the prototype CB_1 _receptor agonist used to compare the present receptor model and verify its correspondence with the previously published docking model [15,16]. As the starting structures for docking, we used the lowest energy conformers of compounds **1 **through **6 **generated from the *SPARTAN *(Version 02, Wavefunction, Inc., Irvine, CA) systematic search employing MMFF. An initial position of the ligands within the receptor pocket was guided by the position of CP55244 determined previously [16]. The docked ligands were inspected for conformations that fit the following two selection rules: (1) there would be an interaction with K3.28(192), which is necessary for high affinity binding and activity for the classical, non-classical, and endocannabinoid classes of compounds [42,43]; and (2) there would be occupation of a similar region of the receptor by the hydrophobic tail of the ligands based on the previously published structure for CP55244 [15].

#### i) By *Glide*/*Prime*

The receptor complex merged with the ligand was subjected to the protein preparation task in *Glide *to check for any incompleteness in the structure. The structure was processed for the grid generation through *Glide*, using van der Waals scaling of the receptor at 0.4 and an electrostatic constraint to K3.28(192). The default size was used for the bounding and enclosing boxes. For the ligand docking stage, van der Waals scaling of the ligand was set at 0.5 and a hydrogen-bond constraint was set to K3.28(192). Of the 50,000 poses that were sampled, 4,000 were taken through minimization (conjugate gradients 1,000) and the 30 structures having the lowest energy conformations were further evaluated for the presence of a H-bonding interaction with K3.28(192) and favourable *Glide *docking score. The four best discrete structures from *Glide *that fit the requirements were each merged with the receptor and processed through the *Prime *task for refining the amino acid side chains. Residues within 10 Å of the ligand were chosen and the Cα movement was set to zero. The refined structure was minimized for residues within 7 Å of the ligand. This newly optimized structure was used for a second cycle of *Glide *docking, using the same grid and run parameters. The best structures from each of the four parallel sequences were compared and the best model structure for the ligand chosen based on number of hydrogen bonds, hydrogen bonding to K3.28(192), binding score, and overall fit to the model for CP55244. This structure was merged with the receptor and minimized again in *Prime *to obtain a final model for the ligand docked within the CB_1 _receptor.

This process was initially applied for compound **1**, the most potent ligand tested in the present study (see Table 1) and then repeated for each of the structurally diverse ligands. Analogs that varied only by the absence of a methyl group (i.e., compound **1 **versus compound **4; **and compound **2 **versus compound **3**) were docked into the model of their methylated counterpart using *Glide *to optimize the conformation of the non-methylated ligand. The final ligand pose chosen was again processed through *Prime *to minimize the complex.

#### ii) By *Affinity*/*SA*

Defining the binding pocket as those residues within 16 Å of the side chain N of K3.28(192), a combination of Monte Carlo and SA simulation procedures was employed to explore docking conformations of the ligands using the *Affinity *module in *InsightII*. During this procedure, the ligand and the side chains of the residues within the defined binding site were allowed to move freely, while other residues were held fixed. The distance between the K3.28(192) side chain N (as the H-bond donor) and the carbonyl O (as the H-bond acceptor) of the ligand was held fixed at 2.1 Å. A set of 100 conformations was obtained from Monte Carlo sampling and subjected to a sequence of energy minimizations: (1) minimization with the TM backbone fixed and the hydrogen bond constrained; and (2) minimization without the hydrogen bond constraint.

The conformation that showed the lowest binding interaction energy (ΔE_bind_) was selected for further sampling by the SA simulation. The distance between the K3.28(192) side chain N and the carbonyl O of the ligand was held fixed at 2.1 Å. The TM backbone was also held fixed, and only the binding pocket residues within 12 Å of the initially bound ligand were allowed to freely move. The *cis *double bond of the ligand was constrained. The protocol for SA involved a short MD of 5 ps at 300 K, heating to 1,500 K by an increase of 200 K per ps, a holding time at 1,500 K for 10 ps, followed by cooling to 300 K by a decrease of 100 K per ps. After holding at 300 K for 5 ps, a sequence of energy minimizations was performed: (1) with the H-bonding and the *cis *double bond constraints; and (2) without these constraints. The final minimized conformer was saved and at the same time used for the next cycle of the SA run. By repeating this procedure, approximately 100 conformers were obtained. Among the lowest ΔE_bind _conformations, several discrete structures were selected. This process was applied for compounds **1**, **2**, **5**, and **6 **(see Table 1). Ligands that varied only by the absence of a methyl group (i.e., compounds **3 **and **4**) were docked into the model of their methylated counterpart and minimized.

### Binding free energy by the linear interaction energy (LIE) method

To determine the most likely binding conformations of the tested compounds **1 **through **6**, the candidate conformations of each compound were examined to obtain the best correlation between their free energy of binding (Δ*G*_bind_) with binding affinity data. The Δ*G*_bind _values were obtained by employing the LIE method [44] implemented in *Liaison *(Schrödinger, Inc. Portland, OR) using the OPLS-2003 force field. In the LIE method, ΔG_bind _= α <ΔU_vdw_> + β <ΔU_elec_> + γ ΔSASA, where <ΔU_vdw_> or <U_elec_> denotes the average change in the van der Waals or the electrostatic interaction energy of the ligand in the free and bound states, respectively, and ΔSASA is the change in the solvent-accessible surface area (SASA). The α, β, and γ terms are adjustable parameters to fit to experimental binding free energy data. To briefly describe the method, a series of hybrid Monte Carlo (HMC) simulations at 300 K were carried out to sample the receptor-ligand complex. The system was initially heated to 300 K in 5 ps and then subjected to a MD simulation for 25 ps. A residue-based cutoff of 12 Å was set for the non-bonding interactions. The non-bonded pair list was updated every 10 fs. The time integration step of 1.0 fs and sampling LIE energies every 10 steps was used. During the MD simulations, all the residues of the protein beyond 12 Å from the bound ligand were frozen. Similarly, the average LIE energies for the ligand were obtained by performing MD simulations as for the receptor-ligand complex. The SASA term required for predicting Δ*G*_bind _was obtained from the cavity term of the implemented Surface Generalized Born continuum solvation model [17].

## Additional files

For the following movies [Additional files 1, 2 and 3], the ligand is in ball-and-stick. The CB_1 _receptor helical backbone is represented in Cα trace format, with TM1 through TM7 colored in red, orange, yellow, green, cyan, blue, and purple, and the extracellular loops colored in magenta. Relevant binding site residues, represented in green color stick, include K3.28(192) (upper left) interacting with the ethanolamide moiety for the H-bond formation, Y5.39(275), F5.42(278), and M6.55(363) that represent the key hydrophobic core residues (lower right) interacting with the heptenyl tail, and F3.25(189) (upper left) and F7.35(379) (lower left) interacting with the benzene ring moiety.

## Competing interests

The author(s) declare that they have no competing interests.

## Authors' contributions

LWP, ACH and J-YS have made substantial contributions to conception and design, analysis and interpretation of data in this manuscript. The biological data are from ACH. LWP generated the *Glide/Prime *analyses, and J-YS generated the *Affinity/SA *analyses and analyzed the PDB files. LWP initiated drafting the manuscript and developed the movies, and LWP, ACH and J-YS continued revising it critically for important intellectual content. All authors have given final approval of the version to be published.

## Supplementary Material

Additional file 1 Compound 1 poses within the CB_1 _receptor having optimal *Affinity *lowest energy values. Click here for file

Additional file 2 Compound 2 poses within the CB_1 _receptor having optimal *Affinity *lowest energy values. Click here for file

Additional file 3 Compound 6 poses within the CB_1 _receptor having optimal *Affinity *lowest energy values. Click here for file

## References

[B1] Devane WA, Hanus L, Breuer A, Pertwee RG, Stevenson LA, Griffin G, Gibson D, Mandelbaum A, Etinger A, Mechoulam R (1992). Isolation and structure of a brain constituent that binds to the cannabinoid receptor. Science.

[B2] Mechoulam R, Ben Shabat S, Hanus L, Ligumsky M, Kaminski NE, Schatz AR, Gopher A, Almog S, Martin BR, Compton DR, . (1995). Identification of an endogenous 2-monoglyceride, present in canine gut, that binds to cannabinoid receptors. Biochem Pharmacol.

[B3] Sugiura T, Kondo S, Sukagawa A, Nakane S, Shinoda A, Itoh K, Yamashita A, Waku K (1995). 2-Arachidonoylglycerol: a possible endogenous cannabinoid receptor ligand in brain. Biochem Biophys Res Commun.

[B4] Hanus L, Abu-Lafi S, Fride E, Breuer A, Vogel Z, Shalev DE, Kustanovich I, Mechoulam R (2001). 2-arachidonyl glyceryl ether, an endogenous agonist of the cannabinoid CB1 receptor. Proc Natl Acad Sci U S A.

[B5] Porter AC, Sauer JM, Knierman MD, Becker GW, Berna MJ, Bao J, Nomikos GG, Carter P, Bymaster FP, Leese AB, Felder CC (2002). Characterization of a novel endocannabinoid, virodhamine, with antagonist activity at the CB1 receptor. J Pharmacol Exp Ther.

[B6] Barnett-Norris J, Guarnieri F, Hurst DP, Reggio PH (1998). Exploration of biologically relevant conformations of anandamide, 2-arachidonylglycerol, and their analogues using conformational memories. J Med Chem.

[B7] Bonechi C, Brizzi A, Brizzi V, Francioli M, Donati A, Rossi C (2001). Conformational analysis of N-arachidonylethanolamide (anandamide) using nuclear magnetic resonance and theoretical calculations. Magnetic Resonance in Chemistry.

[B8] Chen JZ, Han XW, Xie XQ (2005). Preferred conformations of endogenous cannabinoid ligand anandamide. Life Sci.

[B9] Lynch DL, Reggio PH (2005). Molecular dynamics simulations of the endocannabinoid N-arachidonoylethanolamine (anandamide) in a phospholipid bilayer: probing structure and dynamics. J Med Chem.

[B10] Borngraber S, Browner M, Gillmor S, Gerth C, Anton M, Fletterick R, Kuhn H (1999). Shape and specificity in mammalian 15-lipoxygenase active site. The functional interplay of sequence determinants for the reaction specificity. J Biol Chem.

[B11] Gan QF, Browner MF, Sloane DL, Sigal E (1996). Defining the arachidonic acid binding site of human 15-lipoxygenase. Molecular modeling and mutagenesis. J Biol Chem.

[B12] Rowlinson SW, Kiefer JR, Prusakiewicz JJ, Pawlitz JL, Kozak KR, Kalgutkar AS, Stallings WC, Kurumbail RG, Marnett LJ (2003). A novel mechanism of cyclooxygenase-2 inhibition involving interactions with Ser-530 and Tyr-385. J Biol Chem.

[B13] Berglund BA, Fleming PR, Rice KC, Shim JY, Welsh WJ, Howlett AC (2000). Development of a novel class of monocyclic and bicyclic alkyl amides that exhibit CB1 and CB2 cannabinoid receptor affinity and receptor activation. Drug Des Discov.

[B14] Tong W, Collantes ER, Welsh WJ, Berglund BA, Howlett AC (1998). Derivation of a pharmacophore model for anandamide using constrained conformational searching and comparative molecular field analysis. J Med Chem.

[B15] Shim JY, Howlett AC (2004). Steric trigger as a mechanism for CB1 cannabinoid receptor activation. J Chem Inf Comput Sci.

[B16] Shim JY, Welsh WJ, Howlett AC (2003). Homology model of the CB1 cannabinoid receptor: sites critical for nonclassical cannabinoid agonist interaction. Biopolymers.

[B17] Ghosh A, Rapp CS, Friesner RA (1998). Generalized Born model based on a surface integral formulation. J Phys Chem B.

[B18] Mechoulam R, Hanus L, Martin BR (1994). Search for endogenous ligands of the cannabinoid receptor. Biochem Pharmacol.

[B19] Busch-Petersen J, Hill WA, Fan P, Khanolkar A, Xie XQ, Tius MA, Makriyannis A (1996). Unsaturated side chain beta-11-hydroxyhexahydrocannabinol analogs. J Med Chem.

[B20] Fay JF, Dunham TD, Farrens DL (2005). Cysteine residues in the human cannabinoid receptor: only C257 and C264 are required for a functional receptor, and steric bulk at C386 impairs antagonist SR141716A binding. Biochemistry.

[B21] Lu R, Hubbard JR, Martin BR, Kalimi MY (1993). Roles of sulfhydryl and disulfide groups in the binding of CP-55,940 to rat brain cannabinoid receptor. Mol Cell Biochem.

[B22] Gouldson P, Calandra B, Legoux P, Kerneis A, Rinaldi-Carmona M, Barth F, Le Fur G, Ferrara P, Shire D (2000). Mutational analysis and molecular modelling of the antagonist SR 144528 binding site on the human cannabinoid CB(2) receptor. Eur J Pharmacol.

[B23] Shire D, Calandra B, Delpech M, Dumont X, Kaghad M, Le Fur G, Caput D, Ferrara P (1996). Structural features of the central cannabinoid CB1 receptor involved in the binding of the specific CB1 antagonist SR 141716A. J Biol Chem.

[B24] Karnik SS, Sakmar TP, Chen HB, Khorana HG (1988). Cysteine residues 110 and 187 are essential for the formation of correct structure in bovine rhodopsin. Proc Natl Acad Sci U S A.

[B25] Doi T, Molday RS, Khorana HG (1990). Role of the intradiscal domain in rhodopsin assembly and function. Proc Natl Acad Sci U S A.

[B26] Hwa J, Klein-Seetharaman J, Khorana HG (2001). Structure and function in rhodopsin: Mass spectrometric identification of the abnormal intradiscal disulfide bond in misfolded retinitis pigmentosa mutants. Proc Natl Acad Sci U S A.

[B27] Davidson FF, Loewen PC, Khorana HG (1994). Structure and function in rhodopsin: replacement by alanine of cysteine residues 110 and 187, components of a conserved disulfide bond in rhodopsin, affects the light-activated metarhodopsin II state. Proc Natl Acad Sci U S A.

[B28] Karnik SS, Gogonea C, Patil S, Saad Y, Takezako T (2003). Activation of G-protein-coupled receptors: a common molecular mechanism. Trends Endocrinol Metab.

[B29] Murphy JW, Kendall DA (2003). Integrity of extracellular loop 1 of the human cannabinoid receptor 1 is critical for high-affinity binding of the ligand CP 55,940 but not SR 141716A. Biochem Pharmacol.

[B30] Abood ME, Ditto KE, Noel MA, Showalter VM, Tao Q (1997). Isolation and expression of a mouse CB1 cannabinoid receptor gene. Comparison of binding properties with those of native CB1 receptors in mouse brain and N18TG2 neuroblastoma cells. Biochem Pharmacol.

[B31] Ho BY, Zhao J (1996). Determination of the cannabinoid receptors in mouse x rat hybridoma NG108-15 cells and rat GH4C1 cells. Neurosci Lett.

[B32] Okada T, Fujiyoshi Y, Silow M, Navarro J, Landau EM, Shichida Y (2002). Functional role of internal water molecules in rhodopsin revealed by X-ray crystallography. Proc Natl Acad Sci U S A.

[B33] Ulmschneider MB, Tieleman DP, Sansom MS (2005). The role of extra-membranous inter-helical loops in helix-helix interactions. Protein Eng Des Sel.

[B34] Huffman JW, Mabon R, Wu MJ, Lu J, Hart R, Hurst DP, Reggio PH, Wiley JL, Martin BR (2003). 3-Indolyl-1-naphthylmethanes: new cannabimimetic indoles provide evidence for aromatic stacking interactions with the CB(1) cannabinoid receptor. Bioorg Med Chem.

[B35] Friesner RA, Banks JL, Murphy RB, Halgren TA, Klicic JJ, Mainz DT, Repasky MP, Knoll EH, Shelley M, Perry JK, Shaw DE, Francis P, Shenkin PS (2004). Glide: a new approach for rapid, accurate docking and scoring. 1. Method and assessment of docking accuracy. J Med Chem.

[B36] Kenakin T (2002). Drug efficacy at G protein-coupled receptors. Annu Rev Pharmacol Toxicol.

[B37] Melvin LS, Milne GM, Johnson MR, Subramaniam B, Wilken GH, Howlett AC (1993). Structure-activity relationships for cannabinoid receptor-binding and analgesic activity: studies of bicyclic cannabinoid analogs. Mol Pharmacol.

[B38] Vinayagam A, Pugalenthi G, Rajesh R, Sowdhamini R (2004). DSDBASE: a consortium of native and modelled disulphide bonds in proteins. Nucleic Acids Res.

[B39] Chen R, Li L, Weng Z (2003). ZDOCK: an initial-stage protein-docking algorithm. Proteins.

[B40] Chen R, Weng Z (2003). A novel shape complementarity scoring function for protein-protein docking. Proteins.

[B41] Luthy R, Bowie JU, Eisenberg D (1992). Assessment of protein models with three-dimensional profiles. Nature.

[B42] Song ZH, Bonner TI (1996). A lysine residue of the cannabinoid receptor is critical for receptor recognition by several agonists but not WIN55212-2. Mol Pharmacol.

[B43] Chin CN, Lucas-Lenard J, Abadji V, Kendall DA (1998). Ligand binding and modulation of cyclic AMP levels depend on the chemical nature of residue 192 of the human cannabinoid receptor 1. J Neurochem.

[B44] Aqvist J, Marelius J (2001). The linear interaction energy method for predicting ligand binding free energies. Comb Chem High Throughput Screen.

